# Mapping high-grade glioma immune infiltration to 5-ALA fluorescence levels: TCGA data computation, classical histology, and digital image analysis

**DOI:** 10.1007/s11060-023-04406-3

**Published:** 2023-08-06

**Authors:** Alexandra Lang, Raphael L. Jeron, Bastian Lontzek, Barbara Kiesel, Mario Mischkulnig, Anna S. Berghoff, Gerda Ricken, Adelheid Wöhrer, Karl Rössler, Daniela Lötsch-Gojo, Thomas Roetzer-Pejrimovsky, Walter Berger, Johannes A. Hainfellner, Romana Höftberger, Georg Widhalm, Friedrich Erhart

**Affiliations:** 1 Department of Neurosurgery, Medical University of Vienna, Währinger Gürtel 18-20, 1090 Vienna, Austria; 2 Department of Medicine I/Division of Oncology, Medical University of Vienna, Vienna, Austria; 3 Division of Neuropathology and Neurochemistry, Department of Neurology, Medical University of Vienna, Vienna, Austria; 4 Central Nervous System Unit, Comprehensive Cancer Center, Medical University of Vienna, Vienna, Austria; 5 Center for Cancer Research, Medical University of Vienna, Vienna, Austria

**Keywords:** 5-ALA, High-grade glioma, Glioblastoma, Histology, Immune cells, CD8, CD168, CD63, FoxP3, Automated imaging processing

## Abstract

**Purpose:**

Resection of high-grade gliomas has been considerably improved by 5-aminolevulinic acid (5-ALA). However, not all neurobiological properties of 5-ALA are fully understood. Specifically, potential differences in immune infiltration have not been conclusively examined, despite recent reports that immune cells might play a role. Thus, we here provide a systematic mapping of immune infiltration of different 5-ALA fluorescence levels.

**Methods:**

Tumor-associated macrophages (CD68, CD163), cytotoxic T cells (CD8), and regulatory T cells (FoxP3) were quantified via three methods. First, data from The Cancer Genome Atlas (TCGA) of 172 patients was examined for correlations between 5-ALA fluorescence-related mRNA expression signatures and immune markers. Second, as classical histology, 508 stained slides from 39 high-grade glioma patients were analysed semi-quantitatively by two independent reviewers, generating 1016 data points. Third, digital image analysis was performed with automated scanning and algorithm-based cell quantification.

**Results:**

TCGA mRNA data from 172 patients showed a direct, significant correlation between 5-ALA signatures and immune markers (p < 0.001). However, we were not able to confirm this finding in the here studied initial set of 39 patient histologies where we found a comparable immune infiltration in different fluorescence levels. Digital image analysis correlated excellently with standard histology.

**Conclusion:**

With mapping the immune infiltration pattern of different 5-ALA categories, we are adding fundamental basic insights to the field of 5-ALA and glioma biology. The observation that a significant correlation in TCGA data did not fully translate to detectable differences in immune infiltration in first histology data warrants further investigation in larger cohorts.

**Supplementary Information:**

The online version contains supplementary material available at 10.1007/s11060-023-04406-3.

## Introduction

High-grade gliomas are the most common and aggressive malignant brain tumors [1]. Standard-of-care usually starts with maximal safe resection, i.e. fluorescence-guided surgery where 5-aminolevulinic acid (5-ALA) is used as a powerful intraoperative tool [2, 3]. 5-ALA accumulates in malignant tumor tissue where it is metabolized to Protoporphyrin IX (PpIX), shows visible reddish fluorescence under blue excitation light (400–440 nm) and leads to a more effective resection and thereby positively impacts survival [2, 3]. Several aspects of the underlying neurobiology as well as clinical implications of 5-ALA have been elucidated over the last years. For instance, intratumoral regions such as the necrotic core or the peripheral part of the infiltration zone show weak or no visible 5-ALA fluorescence even though they harbour tumor cells [4]. Or, for example, areas with strong 5-ALA fluorescence represent compact tumor regions with high cellularity, mitotic activity, microvessel density and presumably high aggressiveness [5–7]. However, at present, several fundamental facts of 5-ALA biology are not established. Most surprisingly, the immune infiltration pattern of regions with different 5-ALA fluorescence levels have not been conclusively established, despite recent reports that immune cells might be related to fluorescence [8].

In this paper, we provide a first investigation of immune infiltration in regions of different 5-ALA fluorescence levels. We focus on the crucial markers CD8, CD68, CD163 and FoxP3. CD8 is a marker for cytotoxic T cells which play an important role in the killing of malignant cells [9]. CD68+ and CD163+ cells identify macrophages and in line with that also tumor-associated macrophages (TAMs), which are known to be present in high numbers in glioblastomas [9–12]. FoxP3 represents regulatory T cells that act as immune response suppressors [9, 13]. For the quantification of these cell types, three complementary methods are combined to gain a firm understanding of the immune cell infiltration landscape: In-silico analysis of The Cancer Genome Atlas (TCGA) data sets was used to derive a first hypothesis about the interdependence of 5-ALA fluorescence and immune cells. Conventional microscopy analysis of histological slides with semi-quantitative assessment by two independent reviewers was subsequently performed. And finally, automated digital image analysis and immune cell detection were added.

## Methods

### TCGA data analysis

172 patients with available mRNA expression data were retrieved from the two largest available glioblastoma datasets of TCGA. Data were retrieved via the UCSC Xena system on 03/04/2021. Then, 5-ALA fluorescence-associated gene expression signatures were applied to this dataset. In detail, as a proxy for 5-ALA fluorescence, gene expression signatures with known relation to 5-ALA fluorescence were defined based on literature data by Smith et al. and Kim et al. [14, 15]. For the dataset by Smith et al. 77 genes that were at least 2-fold up-regulated in 5-ALA positive samples were combined into a signature. For the dataset by Kim et al. again at least 2-fold up-regulated genes were used, leading to a signature comprising 36 genes. Both signatures were tested for a potential correlation with the chosen immune markers CD8, CD68, CD163 and FoxP3. Pearson correlation coefficients were calculated and regression lines plotted for visualization (GraphPad Prism version 9.3.1.).

### Sample acquisition

We selected 39 patients with resection of a high-grade glioma (CNS WHO grades III and IV) at the Department of Neurosurgery, Medical University of Vienna and preoperative 5-ALA administration from our 5-ALA tissue bank in analogy to previously established protocols [16]. Patients were included if there was at least one 5-ALA negative sample and at least one 5-ALA positive sample and if there was sufficient material available (formalin-fixed, paraffin-embedded tissue blocks, FFPE). For every sample, the specific 5-ALA status as documented during surgery was retrieved. The fluorescence had been rated by the surgeon as *strong, weak* or *negative*, or alternatively simply as *positive* or *negative*. In cases where no further specification of the fluorescence intensity had been made by the surgeon (*positive*), the fluorescence intensity was deemed *positive but unspecified* for the purpose of this study. Solely FFPE blocks with histopathologically confirmed tumor infiltration were chosen. Overall, we included 128 FFPE blocks. The samples were collected following local regulations and the study had been approved by the Ethical Committee of the Medical University of Vienna (EK 419/2008 and 2265/2019).

### Immunohistochemistry

FFPE blocks were cut into 3 μm thick sections and stained on a Dako autostainer system with the following antibodies: CD8 (1:100, Dako Cytomation, M7103), CD68 (1:5000, Dako Cytomation, M0814) and CD163 (1:1000, Novocastra, NCL-L-CD163). After standard deparaffinization with xylene and alcohol, the slides were treated with a sodium citrate solution (pH 6.0) at 95 °C for 20 min to retrieve the antigens, and incubated with the mentioned antibodies for 30 min at room temperature. The Dako detection system (FLEX + Mouse, K8002) was used accordingly. Fox P3 (1:25, BioLegend, 320,116) was stained with an automated Ventana BenchMark staining system and the UltraView Universal DAB detection kit (760−500). Counterstaining of all sections was done manually with hematoxylin.

### Conventional histopathological analysis of stainings

The analysis of the stainings was performed via standard brightfield microscopy. All slides were semi-quantitatively analysed by two independent reviewers, in analogy to well-established protocols [17, 18]. The distribution density of immune cells was graded in the following categories: one (*negative*), two (*sparse*), three (*moderate*) and four (*dense*). All analyses were supervised by the board-certified neuropathologist Dr. Adelheid Wöhrer.

### Digital image analysis via QuPath

Computer-based scanning and automated quantification was performed with QuPath version 0.3.2. Individual thresholds for each antibody staining were set based on quality control stainings to obtain a clear distinction between stained immune cells and nuclei of other cells.

### Statistical analysis

Measured values were arranged according to immune cell marker or vice versa according to 5-ALA fluorescence intensity. For both complementary comparison approaches, statistical testing via ANOVA was performed. Pearson correlation was used for assessing the relation between machine and human measurements. For comparison of two mean values, two-sided T testing was used. p values of less than 0.05 were considered significant. GraphPad Prism version 9.3.1. was the statistical software used.

## Results

### TCGA analysis showed a direct and significant correlation of 5-ALA fluorescence-associated mRNA signatures with immune cell markers

**Fig. 1:**
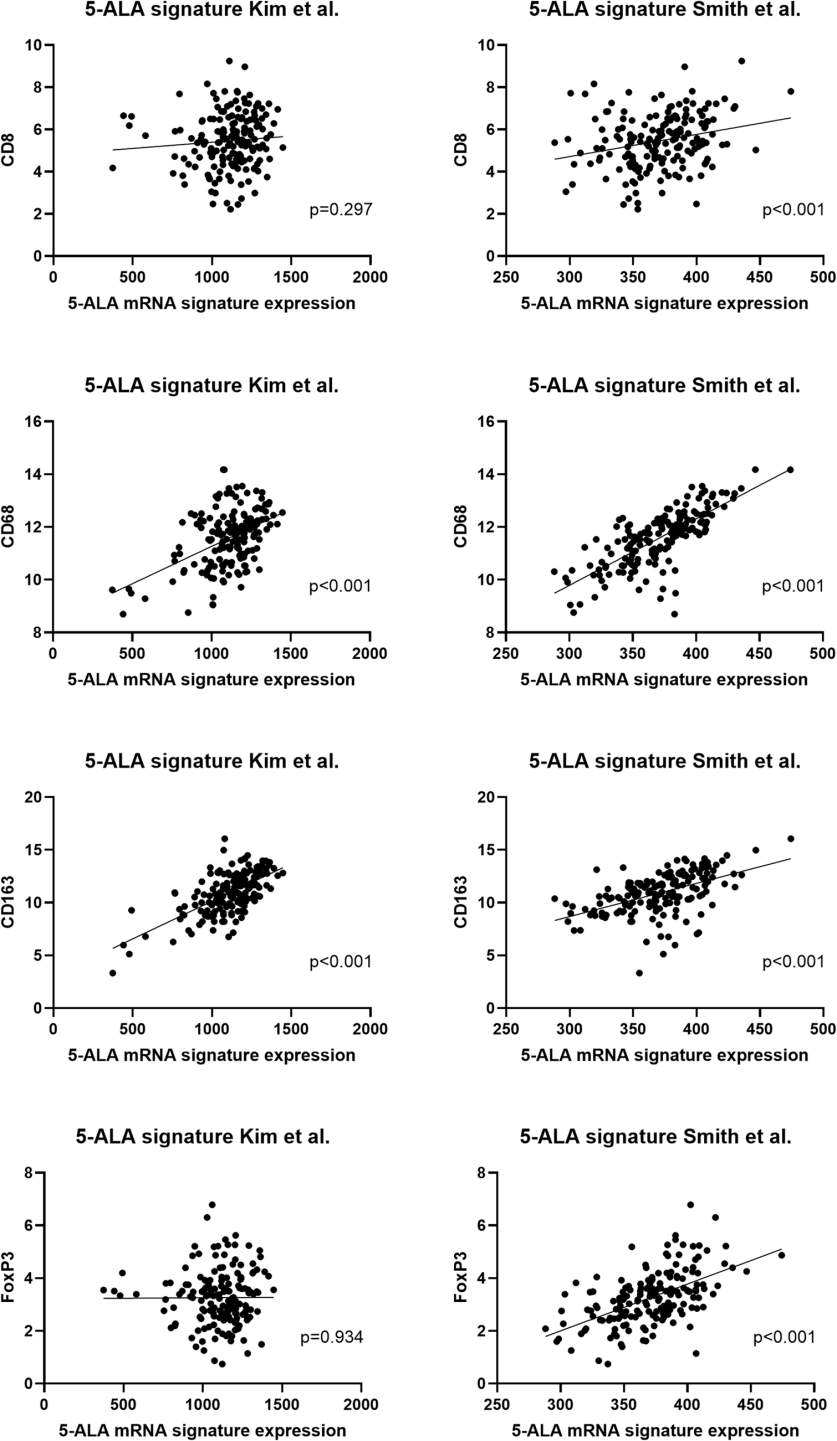
TCGA analysis of correlations between 5-ALA-related gene expression signatures and the respective immune cell markers. The 5-ALA-related signature was derived from two previous studies, i.e. was based on data by Kim et al. and by Smith et al. (n = 172 patients). X and Y axis show log-transformed normalized mRNA expression data in log2 (*norm_value+*1)

As the initial step of investigating the immune cell infiltration pattern in different 5-ALA fluorescence levels, we performed an in-silico analysis based on TCGA data (Fig. 1, n = 172 patients). As a proxy for observable 5-ALA fluorescence, we used two previously published mRNA signatures that had shown a direct association with a fluorescent phenotype [14, 15]. Both signatures were tested for a potential correlation with the four immune cell markers CD8, CD68, CD163, FoxP3 in the two largest available TCGA datasets. As main result we found that CD68 and CD163 mRNA levels correlated significantly with the signature by Kim et al. and all four immune markers correlated significantly with the signature by Smith et al. (Fig. 1).

### 508 stained slides—5-ALA positive and -negative—from 39 high-grade glioma patients were analysed via classical histology

Next, we aimed at substantiating these preliminary TCGA findings with original biological data. For that, we retrospectively recruited a cohort of patients with radiologically suspected glioblastomas. We could include 39 patients with a high-grade glioma into the study (see Supplementary Table). As per the original classification at the time of surgery (2012–2019) 35 of them had a glioblastoma as final diagnosis, two a WHO III high-grade glioma and two a WHO III glioma with transition to glioblastoma. According to the current 2021 WHO classification the IDH status is the decisive criterion. IDH status was known in 37 cases. The vast majority of all patients were IDH-wildtype glioblastoma (95%, i.e. 35/37). 18 patients (46.2%) were female and 21 were male (53.9%). The average age at surgery was 59.6 years. On average, 3.28 samples were retrieved per patient, amounting to a total of 128. Of these, 52 (40.6%) were from tumor regions with no visible fluorescence (i.e. *negative*), 24 (18.8%) with *weak*, 34 (26.6%) with *strong* and 17 (13.3%) with *positive* but unspecified fluorescence (i.e. without further indication of the intensity of the positivity). One sample had no information on 5-ALA fluorescence, due to missing data in the histopathological file. All 128 samples were each stained for the four immune cell markers CD8, CD68, CD163 and FoxP3, leading to an overall amount of 512 stained samples of which four had to be discarded as they did not pass staining quality control and 508 were then used for investigation. With two assessors, a total of 1016 single data points were generated and formed the basis for the subsequent statistical analysis. Interrater agreement was excellent (Supplementary Figs. 1 and 2).

### Across all histology samples, CD68 and CD163 cells were most abundant

**Fig. 2:**
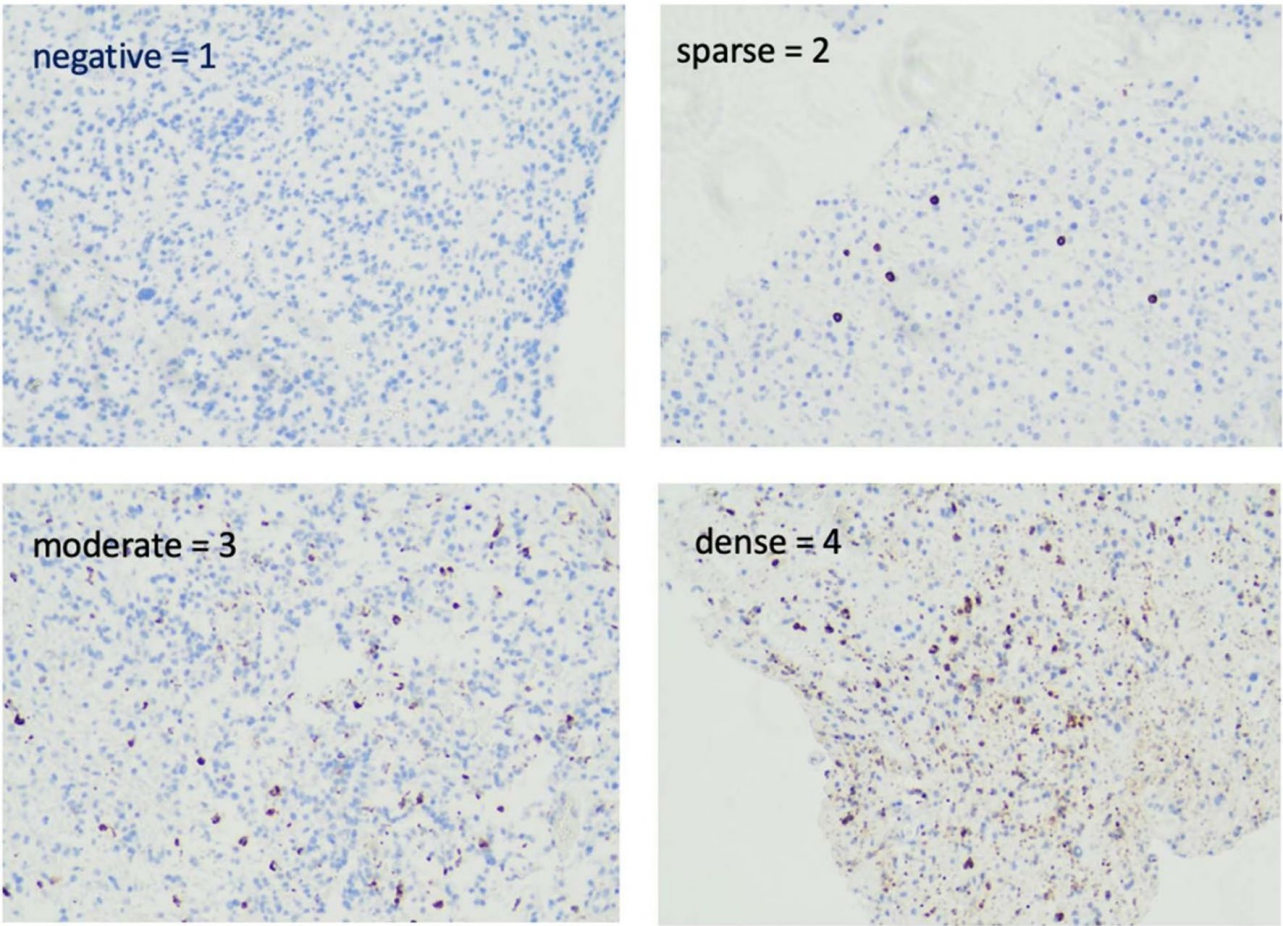
Illustration of the semiquantitative immune cell frequency metric used by the two human assessors. “Negative = 1” shows a slide with negative FoxP3 staining. “Sparse = 2” shows a slide with CD8 staining. “Moderate = 3” shows a slide with CD163. “Dense = 4” shows a slide with CD68

As the first main analytic step, we investigated the general observed immune cell intensity (Fig. 2) across all samples, hence without any distinction according to the 5-ALA fluorescence levels.

We registered that in the vast majority of investigated samples immune cells were present. When interpreting the scored intensity as a numerical value, we observed that CD68 showed the overall highest intensity, followed by CD163 (Supplementary Fig. 3). Hence, both markers that identify TAMs had the generally highest average score. As opposed to that, CD8 and finally FoxP3 showed generally lower average scoring intensities (Supplementary Fig. 3). For both assessors, this difference in the immune cell pattern was highly significant (p < 0.0001).

### In 5-ALA negative and positive high-grade glioma samples the immune cell infiltration pattern seemed the same as per classical histology

As major next part of the analysis, we assessed whether any difference in the immune cell infiltration pattern could be detected in samples with different 5-ALA fluorescence levels. We observed that the distribution pattern looked the same for 5-ALA negative as well as 5-ALA positive samples (Supplementary Fig. 4, assessor A). For CD8 the most frequent intensity across all categories was *sparse* (70.6%). For CD68 it was *dense* (67.7%) and for CD163 the two most frequent intensities were *sparse* (46.5%) and *dense* (36.2%). FoxP3 then showed *sparse* as the most frequent finding for all categories (53.2%). For assessor B the findings were equal (Supplementary Fig. 5).

**Fig. 3:**
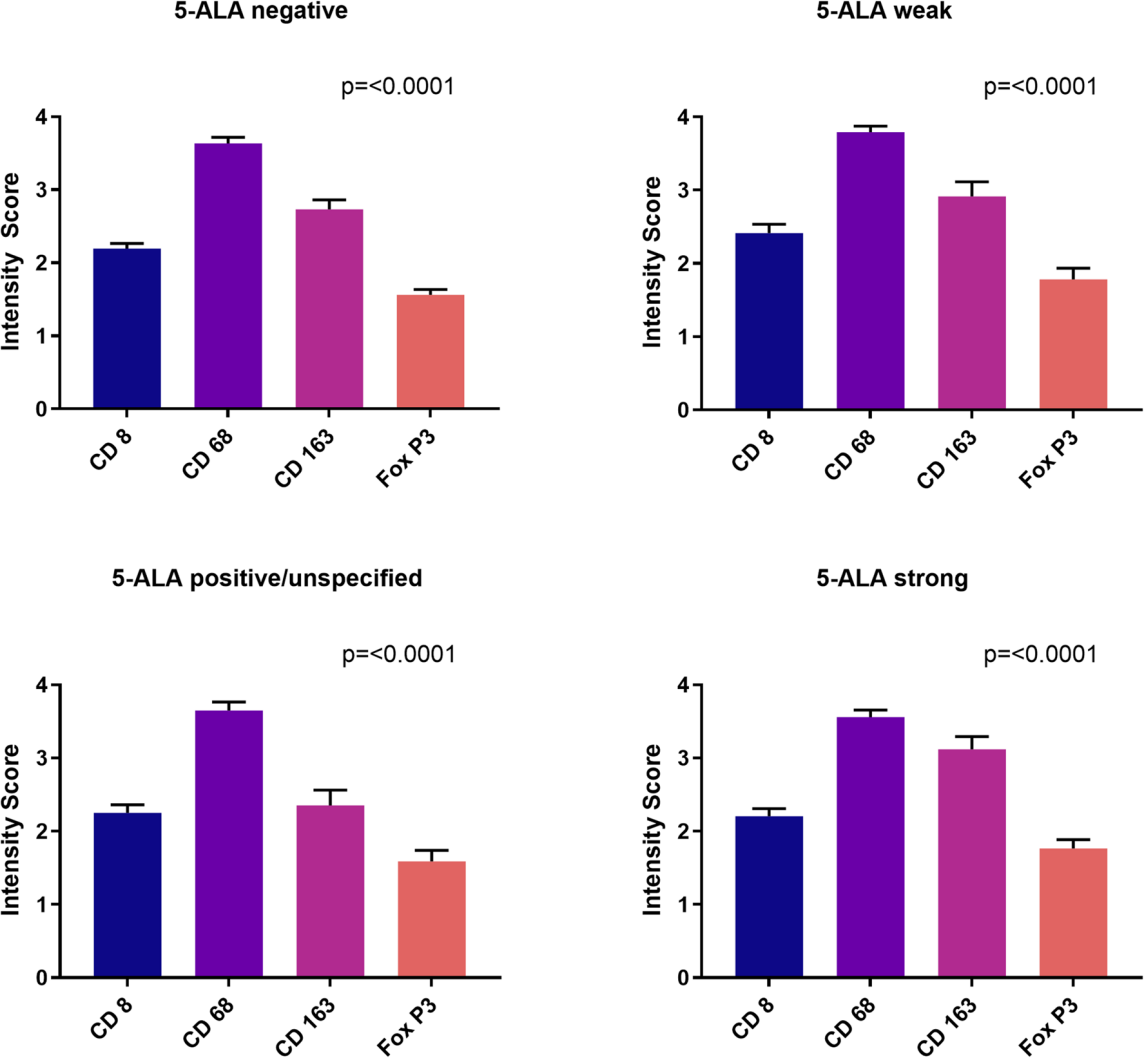
Comparison of the immune cell density for different 5-ALA fluorescence levels. Again, TAM markers (CD68, CD163) have the highest values and they are consistently higher than the markers for cytotoxic T cells (CD8) and regulatory T cells (FoxP3). ANOVA statistical analysis was applied for significance testing

To fully and comprehensively evaluate potential differences between fluorescent and non-fluorescent samples, we also investigated the exact fluorescence levels (*negative*, *weak*, *positive but without further specification* and *strong*). Again, intensity scores were interpreted as numerical values for that analysis. As a result, we observed that in all four 5-ALA fluorescence level categories the overall picture was the same, hence showing an obvious invariance with respect to 5-ALA (Fig. 3, assessor A): CD68 and CD163 had the highest score, followed by CD8 and FoxP3. This finding was highly significant for all categories (p < 0.0001). For assessor B the overall picture was similar (Supplementary Fig. 6).

**Fig. 4:**
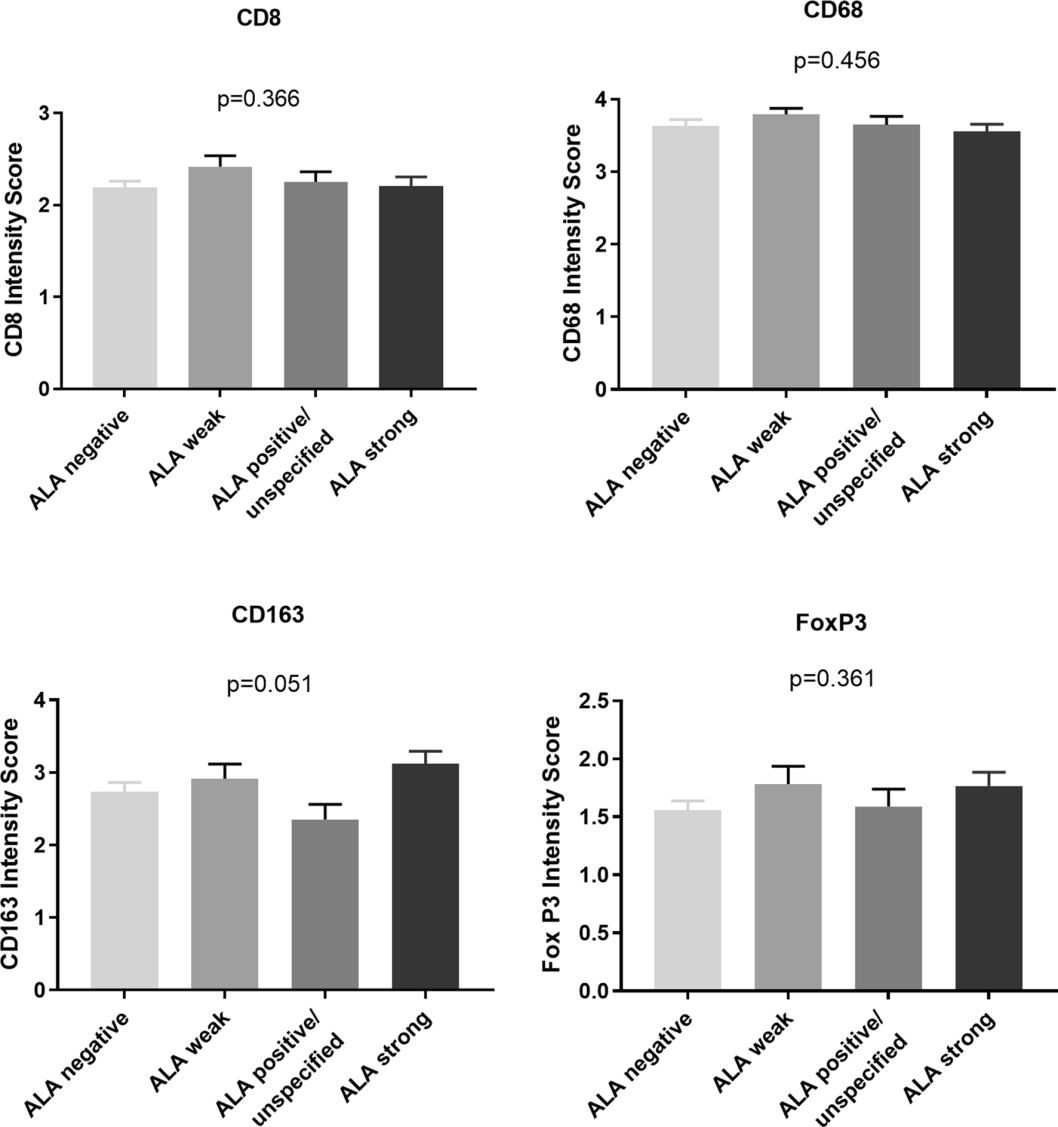
Statistical comparison of 5-ALA fluorescence levels an immune cell intensities, an analysis complementary to Fig. 5. No statistical difference was seen in the immune cell intensity between the various 5-ALA levels. ANOVA statistical analysis was applied for significance testing

Further, to validate this observation from the complimentary statistical angle, we assessed potential differences between 5-ALA fluorescence levels via organizing the data according to single immunological markers and plotting the attributed intensities for the respective 5-ALA fluorescence levels (Fig. 4). Here, for all markers, no statistically significant difference could be registered between the various 5-ALA fluorescence levels, which directly validates the above made observations. Data shown is from assessor A. The results for assessor B were the principally the same—with one single exception, namely CD163 where a minor variation was registered (Supplementary Fig. 7).

### Quantitative automatic digital pathology correlated well with the classical human histopathological analysis

**Fig. 5:**
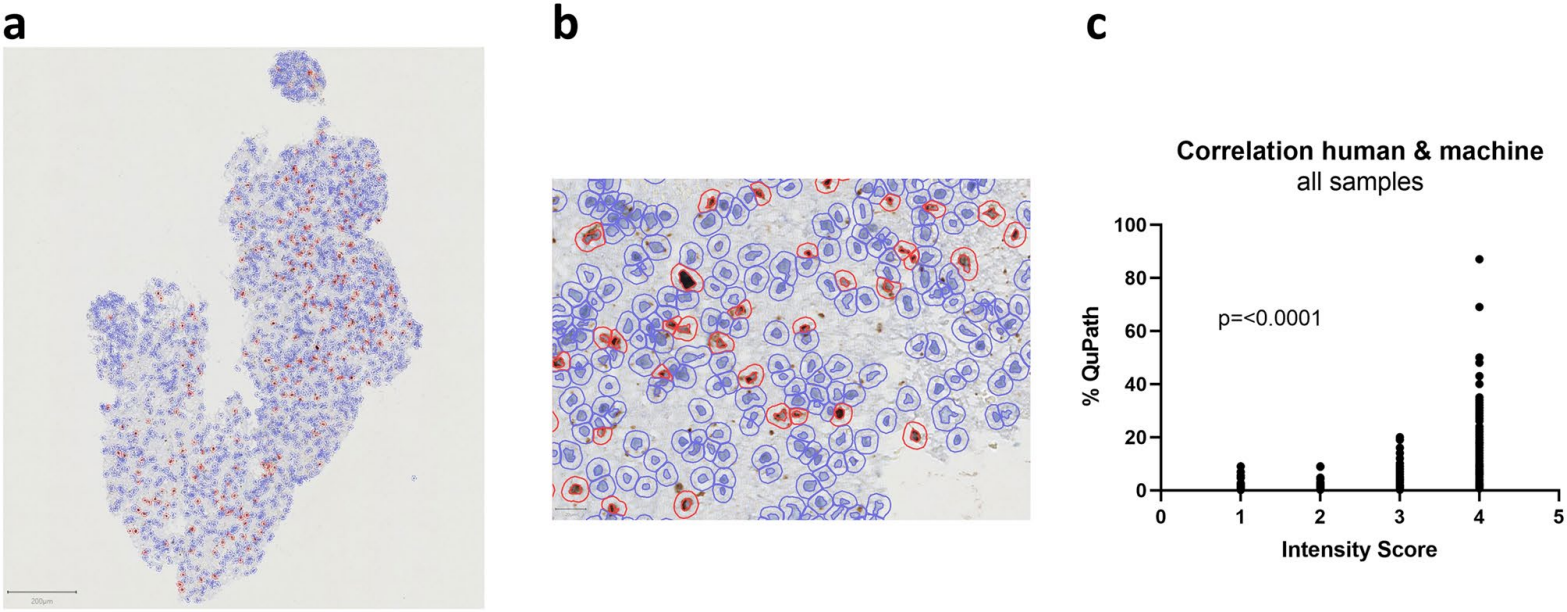
Illustration of the automated digital pathology cell identification and quantification. **a** View of the full slide in one frame. **b** Detail view showing how single cells are segmented and identified. **c** Correlation analysis of all measurements made by a human assessor and made by the algorithm (QuPath). The correlation is highly significant

Finally, to further substantiate the hitherto made findings, we completed the investigation with a quantitative automatic digital pathology analysis [19]. For that purpose, all available slides were fully scanned, digitized and a human-independent algorithm detected all individual cells on the slide and identified the number of cells positive for the respective marker under investigation (Fig. 5a, b). In an initial assessment of general reliability, we tested for an overall correlation of the machine-made findings and the prior human-made observations. In that regard we found a strong and highly significant correlation between the two modes of histopathological scoring (p < 0.0001, Fig. 5c, assessor A).

### Quantitative automatic digital pathology equally concluded that 5-ALA fluorescence levels show a comparable immune cell invasion pattern in the histology samples under investigation

As a last step of the investigation, we analysed the automatic measurements made by the software with respect to the different 5-ALA fluorescence level categories (Supplementary Fig. 8). Again, as was the case in the prior analyses, it was evident that CD68 and CD163 had the overall highest abundance across all samples—and that with a considerably high degree of statistical significance (p < 0.0001, Supplementary Fig. 8). Subsequently, when splitting the data into the respective 5-ALA fluorescence level categories, it was registered that CD68 and CD163 showed the highest frequency in 5-ALA *negative*, *weak* and *strong* samples. For the category 5-ALA *positive but unspecified*, interestingly, only CD68 had the highest count but not CD163. All these findings were highly significant (Supplementary Fig. 8, p < 0.0001).

Again, no statistically significant difference in the immune cell intensity could be found from the complementary statistical angle, i.e. when comparing the 5-ALA categories (Supplementary Fig. 9).

## Discussion

In this study, we performed a systematic analysis of the immune cell infiltration pattern of high-grade glioma tissue with respect to different 5-ALA fluorescence levels.

### 5-ALA fluorescence levels: previous lack of information on immune cell infiltration pattern

In terms of 5-ALA biology several essential observations had previously been made. Lau et al. showed that high-intensity fluorescence areas correspond to cellularity but that lack of fluorescence does not rule out the presence of tumor cells [5]. Bonnin et al. reported that fluorescence has a distinct gene expression, where non-fluorescent samples were consistent with the *neural* glioblastoma subtype [20]. Kim et al. performed quantitative functional proteomics and metabolomics and observed that low NADPH driven by low glutaminase 2 expression is a cause for protoporphyrin IX enrichment [15]. Ross et al. showed that infiltrating tumor margins with low fluorescence express anti-apoptotic and pro-survival proteins [4]. Smith et al. added that invasive glioblastoma areas have an altered stemness profile [21]. Likewise, Smith et al. recently developed 5-ALA based fluorescence-activated cell sorting and used it to further characterize isolated invasive glioblastoma cells [14]. Furthermore, in own previous work we observed that fluorescence intensity is coupled to histological parameters like mitotic activity, nuclear polymorphism, necrotic areas, microvessel density and tumor cell proliferation activity [6]. However, a comprehensive mapping of the immune cell infiltration pattern in samples with different 5-ALA fluorescence levels was still missing so far.

### Present work: mapping high-grade glioma immune infiltration to 5-ALA fluorescence levels

We now closed that knowledge gap and established the immune cell infiltration pattern of different 5-ALA fluorescence levels. As a first observation, we registered that TAMs were generally the most abundant population. This was to be expected as prior research work by others has already firmly concluded that they seem to be the dominant population in glioblastoma [12, 22, 23]. By some accounts, they can make up 30% of the total tumor mass, in rare cases even 50% [24, 25]. In our research, when we performed automated cell counting, we found around 15% TAMs (Supplementary Fig. 8). This refers to the marker CD68. For the marker CD163 we observed markedly lower levels of around 5%. Given that glioblastoma is often regarded as an “immunologically cold” tumor [26], it has to be assumed that the here registered TAMs create an immunosuppressive milieu (especially in this treatment-naïve setting where no specific additional attraction of tumor-infiltrating lymphocytes has apparently occured).

As the main finding of this paper, we registered that the immune cell infiltration pattern was significantly correlated with 5-ALA intensity signatures in TCGA data but was virtually the same in all different 5-ALA fluorescence levels (negative, weak and strong) in the histology samples. In the course of standard histology measurement we did consistently register TAMs as the most frequent population, and we cross-validated this by complementary statistical testing. Conventional semiquantitative assessments as well as automated cell type scoring showed comparable results. For none of the markers, a difference in 5-ALA fluorescence levels was registered. The variance among immune cells—e.g. TAMs most abundant and regulatory T cells least abundant—was similar for all 5-ALA levels and is probably driven by biological factors like the specific immune milieu found in the tumor. In other words, an apparent *invariance* of immune populations with respect to 5-ALA was registered. One minor exception was CD163 as measured by assessor B where a slightly lower value was attributed to the few 5-ALA *positive but unspecified samples* (where the fluorescence intensity had not been further specified by the surgeon)—which we regard as an outlier.

Altogether, even though TCGA data did not readily translate into detectable differences in our histology cohort, it can still be credibly concluded that tumor areas with strong 5-ALA fluorescence seem to have more intense immune infiltration (TCGA data) or at least similar immune infiltration as other tumor regions (histology data)—bearing in mind the usual caveats of statistical testing and further limitations (see below).

Another major learning from this study is the feasibility and reliability of automated, digital image analysis. We here provide a strong argument for expanding its use. High throughput methods like the one used here could be a valuable addition to classical human analysis or could pave the way for standardization of immune cell quantification.

## Limitations

Certain limitations must be taken into account. The data presented is from only one institution and even though the sample size is considerable, it would make sense to add another cohort from another institution to confirm our current findings. This is especially true when considering the apparent disconnect between TCGA results and histology results, that could probably be driven by the relatively smaller sample size of the histology cohort. Alternatively, the obvious discrepancy between TCGA and histology might be traced back to fluorescence measurement challenges, like the potentially minor nature of the actual differences between the fluorescence classes – making them undetectable. Similarly, it could be attributed to the lack of a clear-cut classification of the fluorescence from the samples. Therefore, for future research, it would be advisable to use truly quantitative fluorescence measures during surgery like spectroscopic probes that have recently been introduced [27]. What is more, despite our targeted selection of the most important immune cells to measure, we could only cover a subset of all immune populations found in gliomas. Further continuative studies could e.g. include myeloid-derived suppressor cells or could better sub-characterize immune cells (e.g. M1 versus M2 TAMs).

## Conclusions

With the work performed here, we provide a first mapping of high-grade glioma immune infiltration to 5-ALA fluorescence levels via three complementary technologies—TCGA data computation, classical histology, and digital image analysis. Tumor areas with strong 5-ALA fluorescence seem to have more intense immune infiltration (TCGA data) or at least similar immune infiltration as other tumor regions (histology data). Sample size and subjective assessment of 5-ALA fluorescence by the surgeon could be drivers why a significant correlation in TCGA data did not readily translate to detectable differences in histology data.

Further, independently of 5-ALA intensity, we generally confirm that TAMs are the dominant population in high-grade gliomas and that T cells are less frequent. And finally, with the work done here, we illustrate the usability of high-throughput digital image analysis in immunooncology.

## Supplementary Information

Below is the link to the electronic supplementary material.

Supplementary material 1 (PDF 840.0 kb)

## References

[1] Tan AC, Ashley DM, López GY, Malinzak M, Friedman HS, Khasraw M (2020). Management of glioblastoma: state of the art and future directions. CA Cancer J Clin.

[2] Hadjipanayis CG, Widhalm G, Stummer W (2015). What is the surgical benefit of utilizing 5-aminolevulinic acid for fluorescence-guided surgery of malignant gliomas?. Neurosurgery.

[3] Stummer W (2006). Fluorescence-guided surgery with 5-aminolevulinic acid for resection of malignant glioma: a randomised controlled multicentre phase III trial. Lancet Oncol.

[4] Ross JL (2017). 5-Aminolevulinic acid guided sampling of glioblastoma microenvironments identifies pro-survival signaling at infiltrative margins. Sci Rep.

[5] Lau D (2016). A prospective phase II clinical trial of 5-aminolevulinic acid to assess the correlation of intraoperative fluorescence intensity and degree of histologic cellularity during resection of high-grade gliomas. J Neurosurg.

[6] Kiesel B (2018). Systematic histopathological analysis of different 5-aminolevulinic acid–induced fluorescence levels in newly diagnosed glioblastomas. J Neurosurg.

[7] Mischkulnig M (2022). The impact of heme biosynthesis regulation on glioma aggressiveness: correlations with diagnostic molecular markers. Front Mol Neurosci.

[8] Ryabova A (2022). Detection of changes in macrophage polarization as a result of 5-aminolevulinic acid photodynamic therapy using fluorescence-lifetime imaging microscopy. Photonics.

[9] Gieryng A, Pszczolkowska D, Walentynowicz KA, Rajan WD, Kaminska B (2017). Immune microenvironment of gliomas. Lab Invest.

[10] Buonfiglioli A, Hambardzumyan D (2021). Macrophages and microglia: the cerberus of glioblastoma. Acta Neuropathol Commun.

[11] Urbantat RM (2021). Tumor-associated microglia/macrophages as a predictor for survival in glioblastoma and temozolomide-induced changes in cxcr2 signaling with new resistance overcoming strategy by combination therapy. Int J Mol Sci.

[12] Parney IF, Waldron JS, Parsa AT (2009). Flow cytometry and in vitro analysis of human glioma-associated macrophages: laboratory investigation. J Neurosurg.

[13] Zheng Y, Rudensky AY (2007). Foxp3 in control of the regulatory T cell lineage. Nat Immunol.

[14] Smith SJ (2020). Metabolism-based isolation of invasive glioblastoma cells with specific gene signatures and tumorigenic potential. Neurooncol Adv.

[15] Kim S (2017). Glutaminase 2 expression is associated with regional heterogeneity of 5-aminolevulinic acid fluorescence in glioblastoma. Sci Rep.

[16] Mischkulnig M (2022). Heme biosynthesis factors and 5-ALA Induced fluorescence: analysis of mRNA and protein expression in fluorescing and non-fluorescing gliomas. Front Med (Lausanne).

[17] Berghoff AS (2014). Programmed death ligand 1 expression and tumor-infiltrating lymphocytes in glioblastoma. Neuro Oncol.

[18] Berghoff AS (2017). Correlation of immune phenotype with IDH mutation in diffuse glioma. Neuro Oncol.

[19] Bankhead P (2017). QuPath: open source software for digital pathology image analysis. Sci Rep.

[20] Bonnin DAA (2020). Characterizing the heterogeneity in 5-aminolevulinic acid-induced fluorescence in glioblastoma. J Neurosurg.

[21] Smith SJ, Diksin M, Chhaya S, Sairam S, Estevez-Cebrero MA, Rahman R (2017). The invasive region of glioblastoma defined by 5ALA guided surgery has an altered cancer stem cell marker profile compared to central Tumour. Int J Mol Sci.

[22] Quail DF, Joyce JA (2017). The microenvironmental landscape of brain tumors. Cancer Cell.

[23] Graeber MB, Scheithauer BW, Kreutzberg GW (2002). Microglia in brain tumors. GLIA.

[24] Lapa C (2015). Tumor-associated macrophages in glioblastoma multiforme-A suitable target for somatostatin receptor-based imaging and therapy?. PLoS ONE.

[25] Morimura T (1990). Monocyte subpopulations in human gliomas: expression of Fc and complement receptors and correlation with tumor proliferation. Acta Neuropathol.

[26] Grabowski MM (2021). Immune suppression in gliomas. J Neurooncol.

[27] Widhalm G (2019). The value of visible 5-ALA fluorescence and quantitative protoporphyrin IX analysis for improved surgery of suspected low-grade gliomas. J Neurosurg.

