# Supplementary material for: Mapping high-grade glioma immune infiltration to 5-ALA fluorescence levels: TCGA data computation, classical histology, and digital image analysis
Source: J Neurooncol. 2023 Aug 6;164(1):211–20. doi: 10.1007/s11060-023-04406-3 (PMC10462498; doi:10.1007/s11060-023-04406-3)
Supplement: Supplementary file 1 — Supplementary material 1 (PDF 840.0 kb) [file 11060_2023_4406_MOESM1_ESM.pdf]

Journal of Neurooncology

Mapping high-grade glioma immune infiltration to 5-ALA fluorescence levels: TCGA data computation, classical histology, and digital image analysis

Alexandra Lang[1,4], Raphael L. Jeron[1], Bastian Lontzek[1], Barbara Kiesel[1,4], Mario Mischkulnig[1,4], Anna S. Berghoff[2,4], Gerda Ricken[3,4], Adelheid Wöhrer[3,4], Karl Rössler[1,4], Daniela Lötsch-Gojo[1,4], Thomas Roetzer-Pejrimovsky[3,4], Walter Berger[5], Johannes A. Hainfellner[3,4], Romana Höftberger[3,4], Georg Widhalm[1,4*], Friedrich Erhart[1,4]

1 Department of Neurosurgery, Medical University of Vienna, Austria
2 Department of Medicine I/Division of Oncology, Medical University of Vienna, Austria
3 Division of Neuropathology and Neurochemistry, Department of Neurology, Medical University of Vienna, Austria
4 Central Nervous System Unit, Comprehensive Cancer Center, Medical University of Vienna, Austria
5 Center for Cancer Research, Medical University of Vienna, Austria

* Corresponding Author:

Georg Widhalm
georg.widhalm@meduniwien.ac.at

Supplementary Figure 1

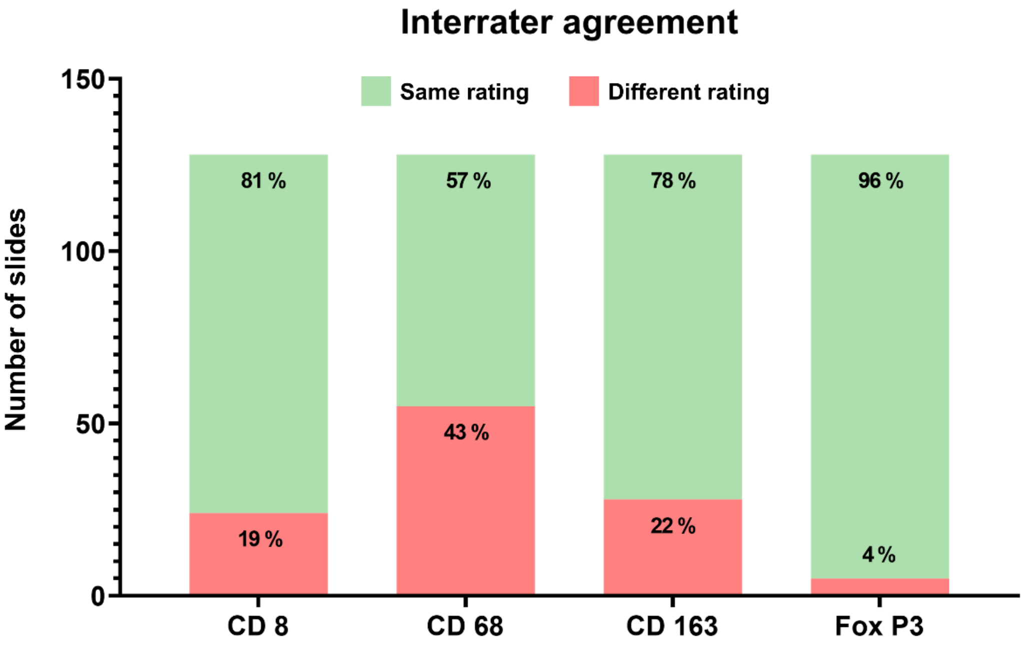

Supplementary Figure 2

Correlation coefficient analysis of the different assessments made by the two independent assessors.

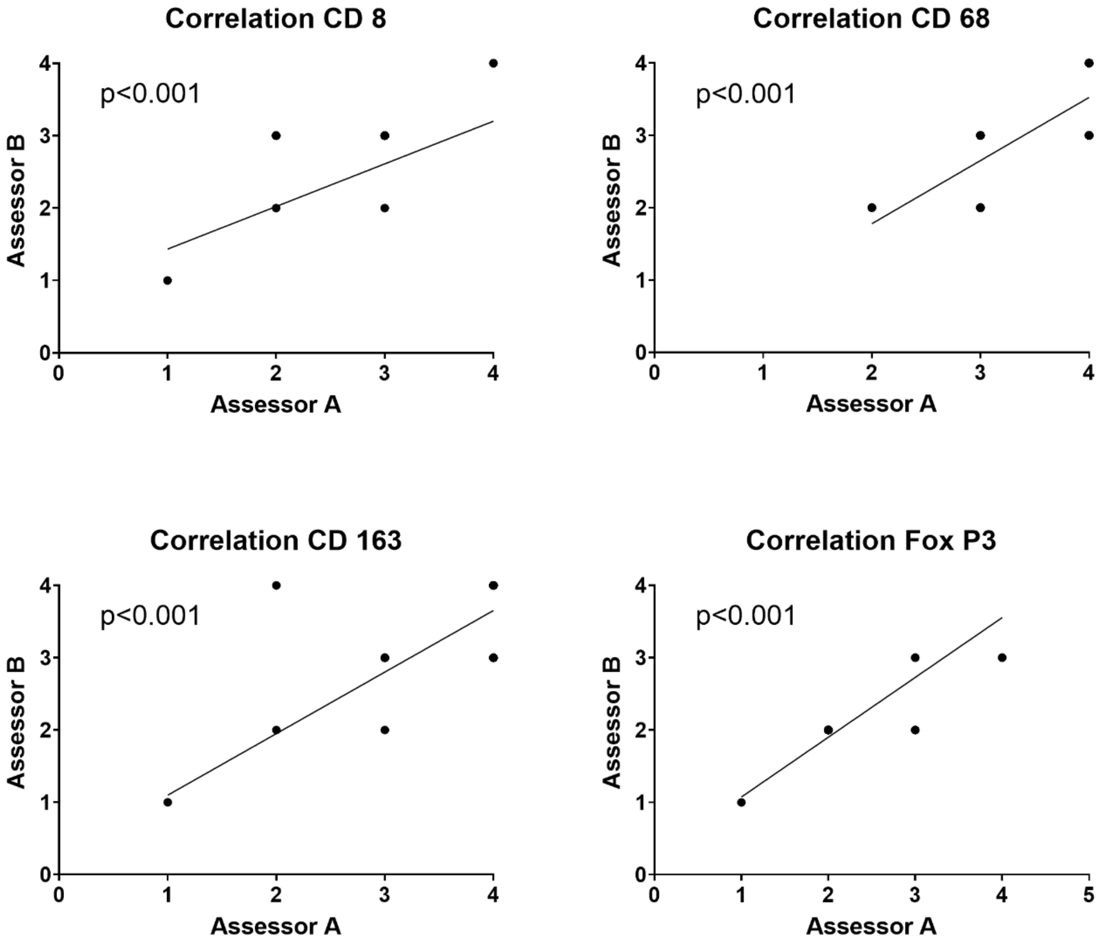

Supplementary Figure 3

Intensity scores for the respective histological markers, as judged by the two assessors. CD68 and CD163 identify tumor-associated macrophages (TAM). CD8 identifies cytotoxic T cells and Fox P3 identifies regulatory T cells. Attached you can find the summary table of all gradings of assessor A and B with calculated mean values and standard deviation.

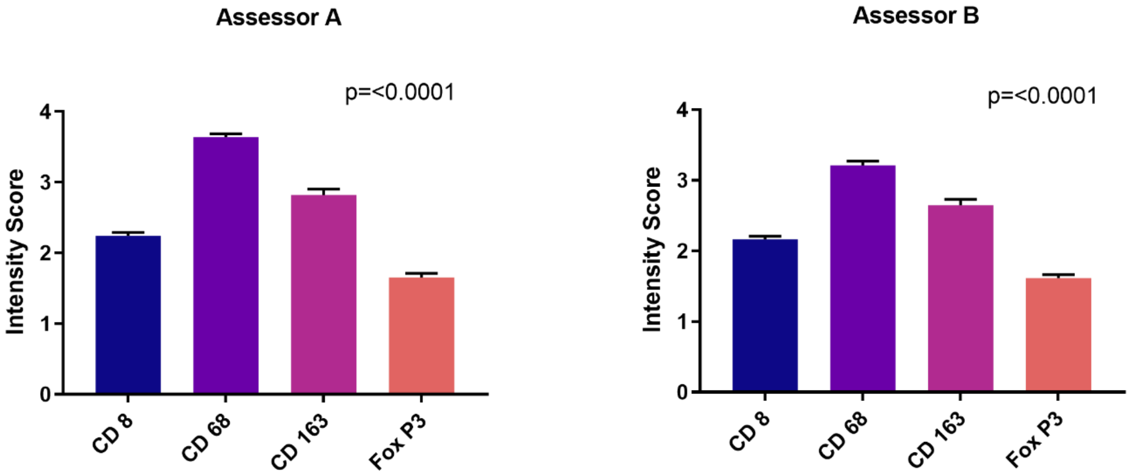

	Ass. A	Ass. B	Ass. A	Ass. B	Ass. A	Ass. B	Ass. A	Ass. B
	CD 8	CD 8	CD 68	CD 68	CD 163	CD 163	Fox P3	Fox P3
**Mean** ± SD	**2,24** ± 0,53	**2,17** ± 0,48	**3,64** ± 0,54	**3,21** ± 0,68	**2,82** ± 0,98	**2,65** ± 0,93	**1,66** ± 0,64	**1,62** ± 0,55
	Ass. A	Ass. B	Ass. A	Ass. B	Ass. A	Ass. B	Ass. A	Ass. B
Patient	CD 8	CD 8	CD 68	CD 68	CD 163	CD 163	Fox P3	Fox P3
1	2	2	4	4	4	4	2	2
	3	2	4	4	4	4	2	2
	2	2	4	4	2	2	1	1
2	1	1	4	3	2	2	1	1
	1	1	4	4	3	3	1	1
	2	2	4	3	2	2	2	2
	2	2	4	4	2	2	1	1
	2	3	4	4	3	2	2	2
3	2	3	3	2	2	2	1	1
	3	3	3	3	3	2	2	2

	2	2	3	3	2	2	1	1
	2	2	4	4	2	2	2	2
	2	2	4	3	2	2	1	1
4	2	3	3	2	1	1	2	2
	2	2	3	2	2	2	1	1
	2	2	4	3	2	2	2	2
5	2	2	4	3	4	3	2	2
	1	1	4	4	2	2	1	1
6	3	3	3	3	4	4	2	2
	3	3	3	3	2	4	2	2
	3	3	4	3	3	3	1	1
	2	2	4	3	3	2	1	1
	2	2	4	4	2	2	1	1
	2	3	4	4	2	2	1	1
7	3	2	3	2	2	2	2	2
	2	2	3	3	4	3	2	2
	3	3	4	4	4	3	2	2
	3	2	3	3	2	2	2	2
	2	2	4	4	3	2	2	2
	2	3	4	4	4	3	2	2
	3	3	4	4	2	2	2	2
	3	2	4	4	2	2	2	2
8	2	2	4	4	4	4	1	1
	2	2	3	3	4	4	1	1
	2	2	4	4	4	4	2	2
	2	2	4	4	2	2	1	1
	2	2	3	3	3	3	1	1
9	2	2	3	3	2	2	1	1
10	3	3	4	4	4	4	3	3
	2	2	4	4	4	4	1	1
	2	2	4	3	4	4		
11	3	2	4	4	3	3	2	2
	2	3	4	4	4	4	2	2
	2	2	4	3	2	2	2	2
12	2	2	4	4	2	2	2	2
	2	2	4	4	4	4	1	1
	3	3	3	3	3	2	1	1
13	2	2	3	2	4	4	1	1
	2	2	3	3	3	3	2	2
	2	2	2	2	2	2	2	2
14	2	2	4	3	4	4	2	2
15	2	2	4	3	2	2	1	1
	2	2	4	4	3	2	1	1
	2	2	4	3	3	2	2	2
16	3	3	3	3	4	4	2	2

	3	3	3	2	4	4	1	1
	3	2	4	4	2	2	2	2
17	2	2	3	3	4	4	2	2
	2	2	3	3	4	3	1	1
	2	2	4	3	4	3	1	1
18	2	2	4	3	2	2	1	1
	2	2	2	2	2	2	1	1
19	2	2	4	3	2	2	1	1
	3	2	4	3	2	2	2	2
	2	2	3	3	2	2	1	1
20	2	2	4	3	1	1	1	1
	2	2	3	2	2	2	1	1
21	2	2	3	3	2	2	2	2
	2	2	3	2	2	2	1	1
			3	2	3	2	1	1
22	3	3	4	4	4	4	2	2
	2	2	4	4	4	4	1	1
	3	2	4	4	2	2	2	2
	3	2	4	4	2	2	2	2
	2	2	4	4	2	2	1	1
23	3	2	4	3	1	1	2	2
	2	2	4	3	2	2	2	2
	2	2	4	3	2	2	2	2
	2	2	4	3	1	1	1	1
	3	2	4	3	2	2		
24	2	2	3	2	2	2	1	1
	2	2	3	2	2	2	1	1
	3	2	4	3	2	2	2	2
	2	2	4	3	2	2	2	2
	2	2	4	3	2	2	2	2
25	3	2	4	3	4	4	1	1
	2	2	4	3	4	3	1	1
	3	3	4	3	4	3	3	2
26	2	2	3	2	2	2	2	2
	2	2	3	2	2	2	2	2
27	2	2	4	3	2	2	2	2
	2	2	4	3	2	2	1	1
	2	2	2	2	2	2	1	1
	2	2	2	2	1	1	1	1
28	3	2	4	3	4	4	3	2
	2	2	4	3	2	2	1	1
29	2	3	4	4	4	4	2	2
	2	2	4	4	4	4	2	2
30	2	2	4	4	4	3	2	2
	2	2	4	4	4	4		

	2	2	4	4	4	3	2	2
	2	2	4	3	3	2	2	2
31	2	2	3	3	2	2	1	1
	2	2	3	3	2	2	2	2
	2	2	3	3	2	2	2	2
32	3	2	4	4	4	4	3	2
	3	2	4	3	2	2	2	2
33	2	2	3	3	3	2	1	1
34	1	1	3	2	4	3	1	1
35	2	2	4	3	4	4	2	2
	3	2	4	4	4	4	2	2
	2	2	4	3	4	4	2	2
36	2	2	3	2	2	2	2	2
	2	2	3	3	2	2	2	2
	2	2	3	3	2	2	1	1
37	2	2	3	3	2	2	2	2
	4	4	4	4	3	2	4	3
	3	3	4	4	3	2	3	3
38	4	4	4	4	4	4	4	3
	2	2	4	3	4	4	2	2
	2	2	4	4	2	2	2	2
	2	2	4	3	3	3	2	2
39	2	2	4	3	4	4	2	2
	3	3	4	4	4	4	2	2
	2	2	4	4	4	4	2	2
	2	2	4	4	4	3	1	1
	2	2	4	3	4	4	1	1
	2	2	4	4	4	4	2	2

Supplementary Figure 4

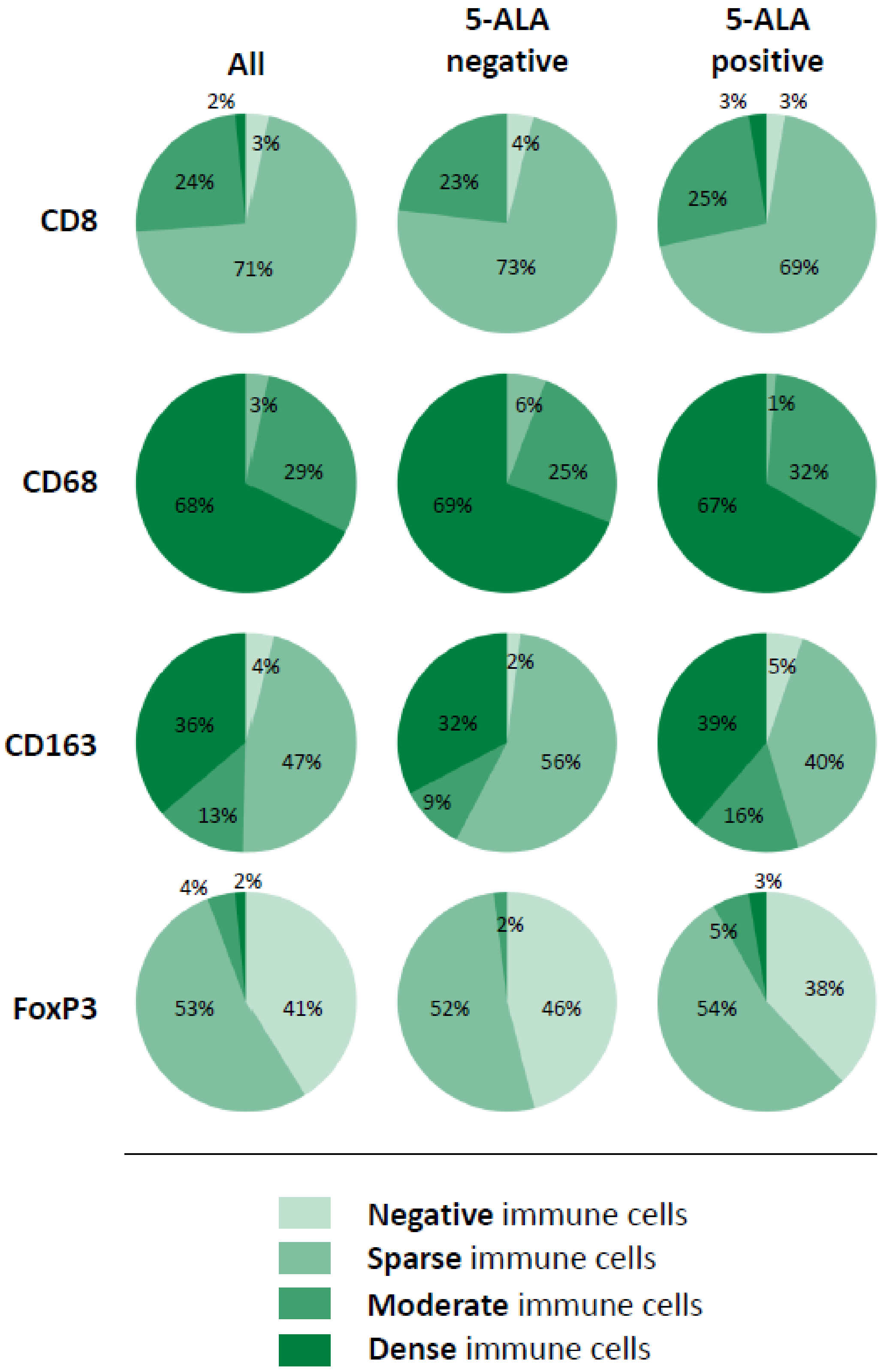

Supplementary Figure 5

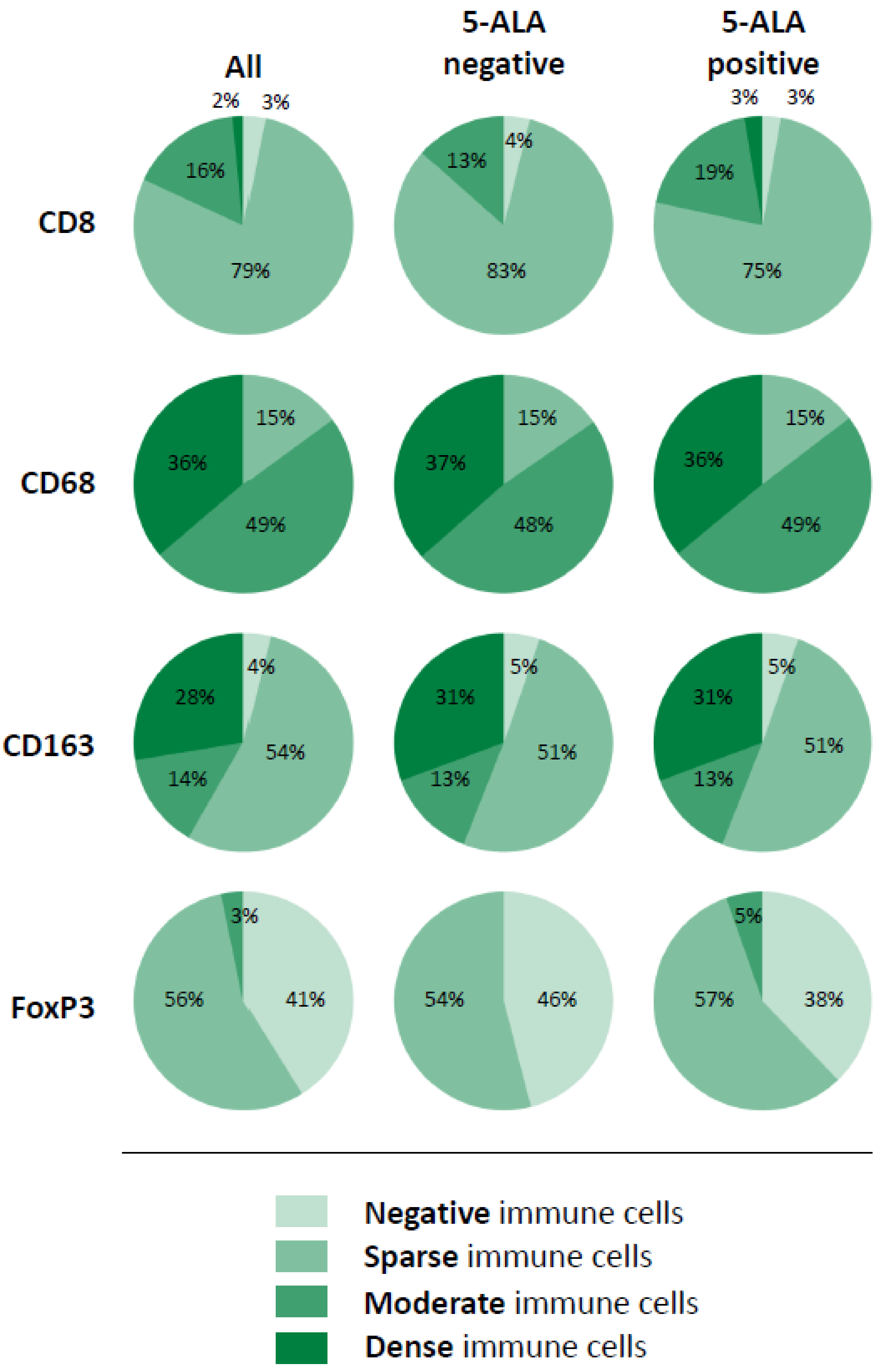

Supplementary Figure 6

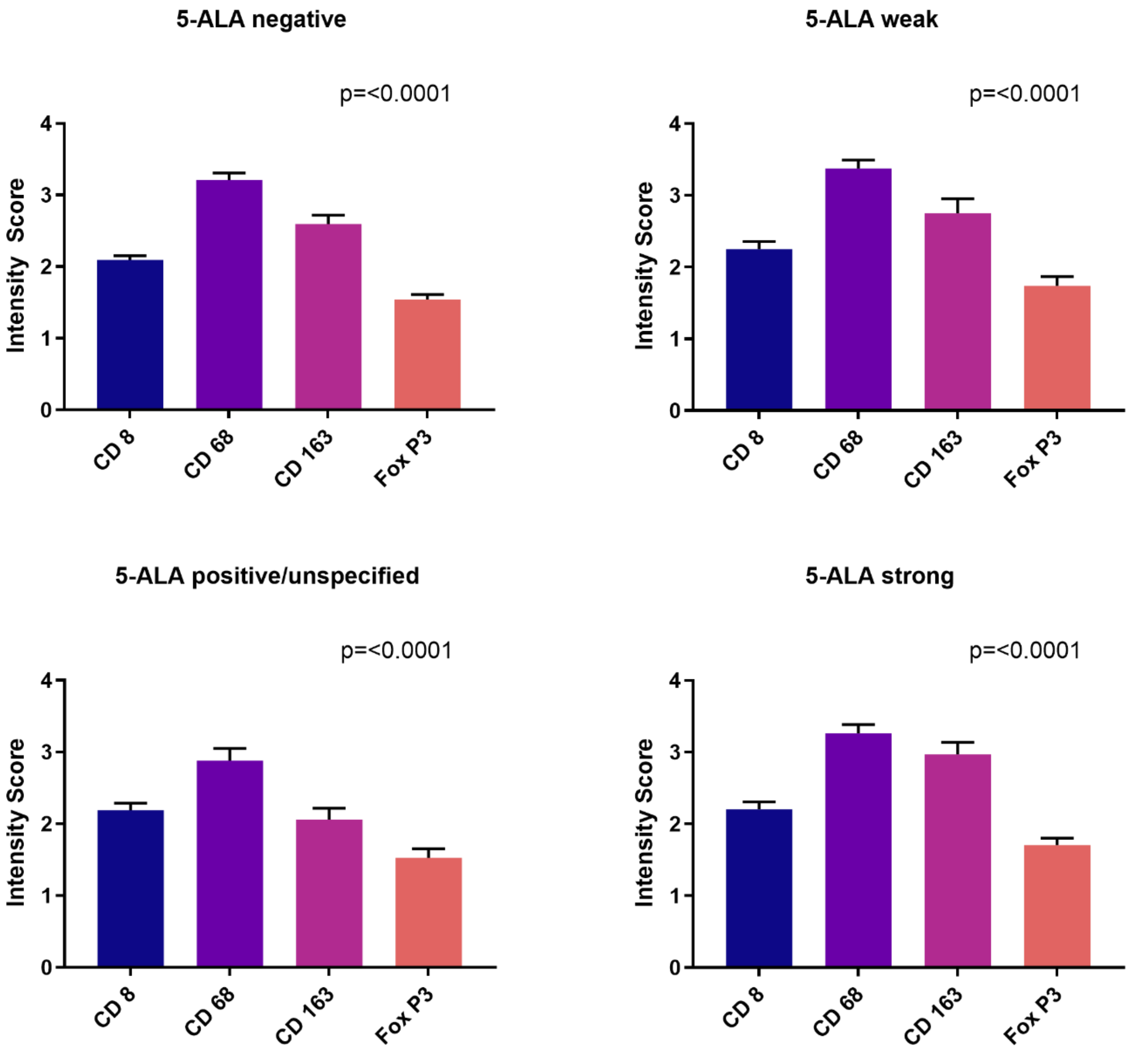

Supplementary Figure 7

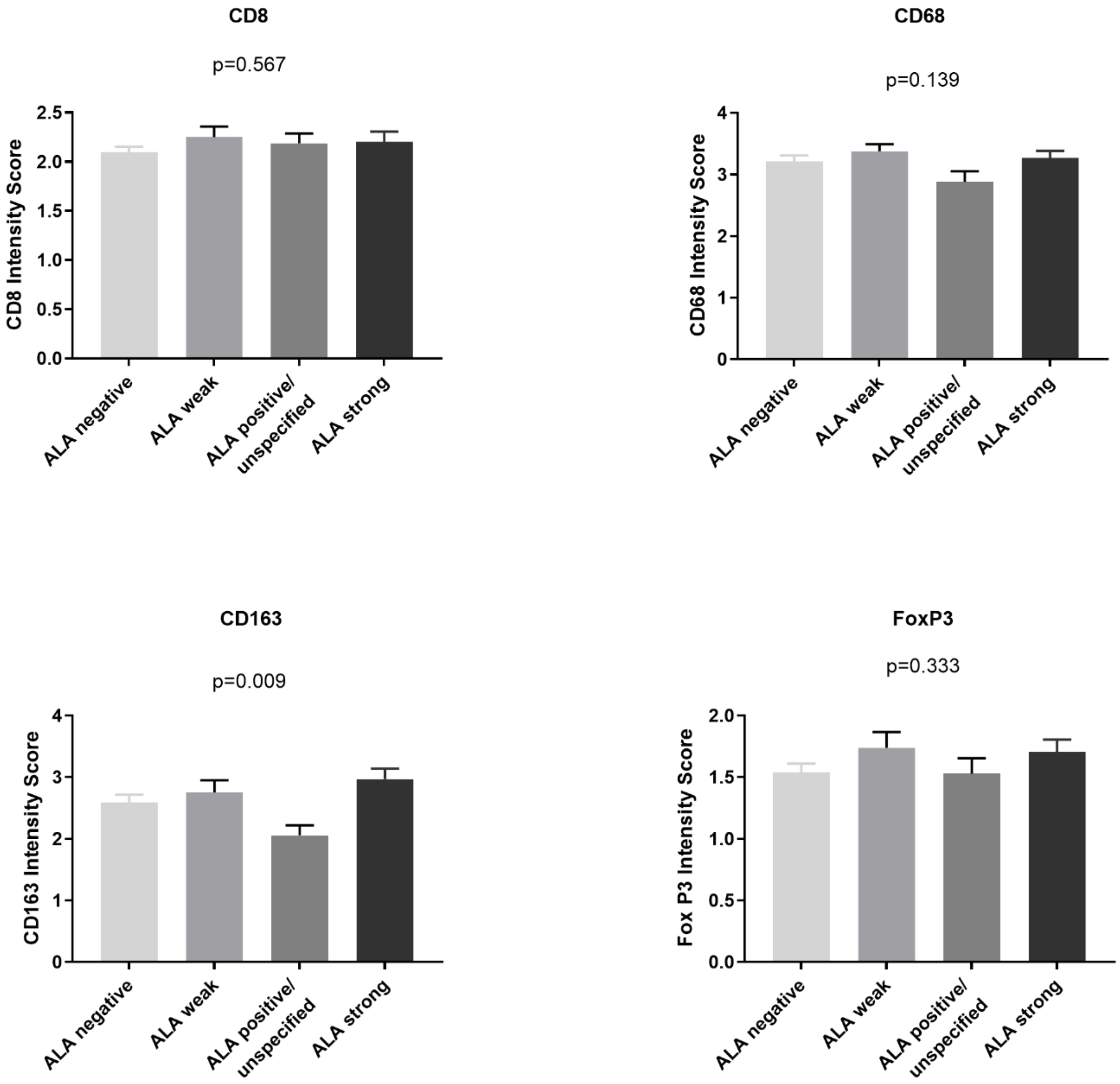

Supplementary Figure 8

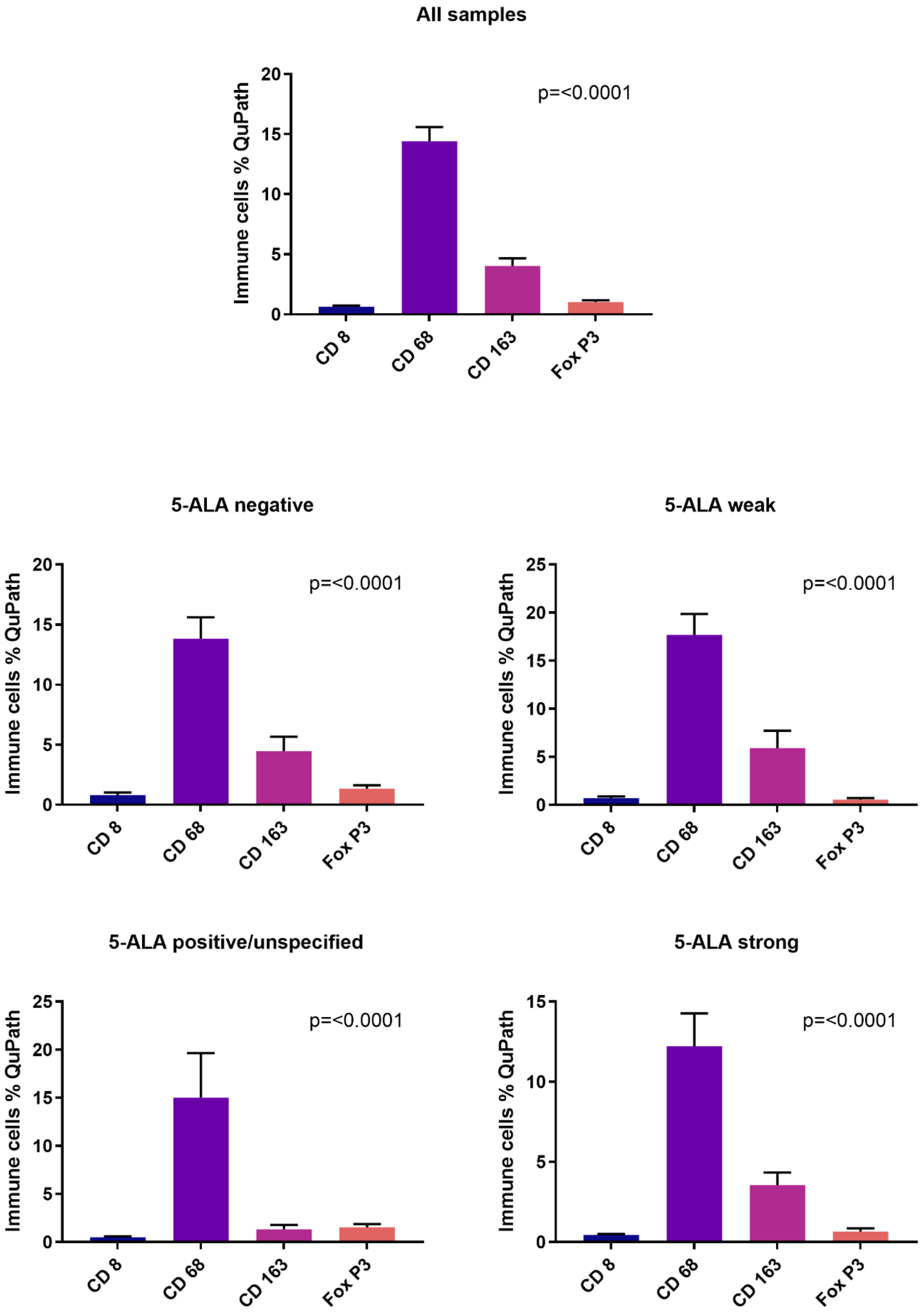

Supplementary Figure 9

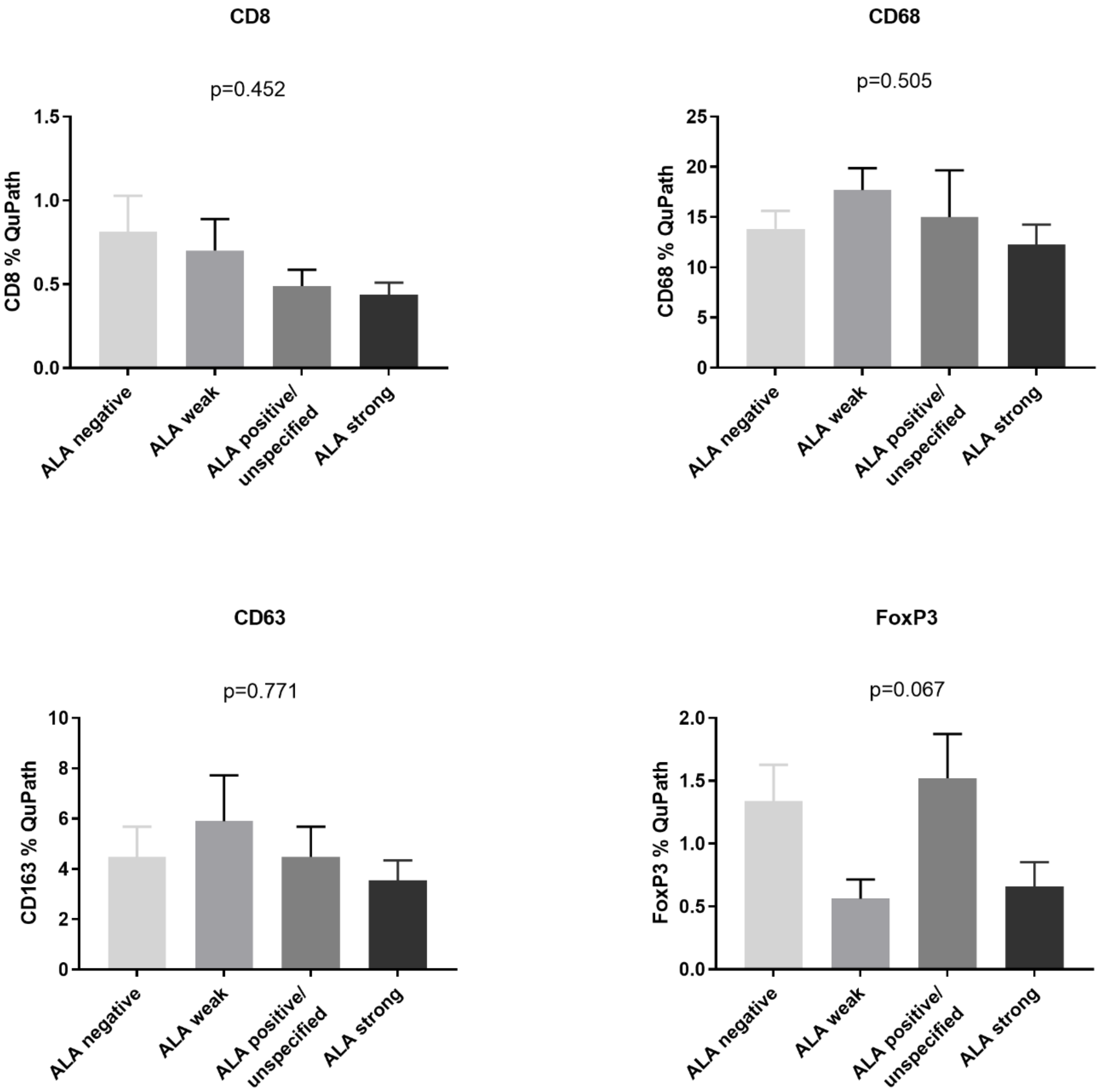

Supplementary Table

Patients' characteristics

Patient	Sex	Age	IDH/WT	Diagnose
1	M	72	WT	GBM, WHO IV
2	F	45	IDH1-mutiert	GBM, WHO IV
3	F	38	IDH1-mutiert	GBM, WHO IV
4	F	60	WT	GBM, WHO IV
5	F	40	IDH1-mutiert	GBM, WHO IV
6	M	53	?	Anaplastic Astrocytoma/GBM
7	M	40	WT	GBM, WHO IV
8	F	80	WT	GBM, WHO IV
9	M	42	?	GBM, WHO IV
10	M	68	WT	GBM, WHO IV
11	F	72	?	GBM, WHO IV
12	M	70	WT	GBM, WHO IV
13	M	46	WT	GBM, WHO IV
14	F	57	WT	GBM, WHO IV
15	F	55	WT	GBM, WHO IV
16	M	54	WT	GBM, WHO IV
17	M	79	WT	GBM, WHO IV
18	M	71	WT	GBM, WHO IV
19	F	58	WT	GBM, WHO IV
20	M	29	WT	GBM, WHO IV
21	M	62	WT	GBM, WHO IV
22	M	52	WT	Anaplastic Astrocytoma/GBM(WHO III-IV)
23	F	68	WT	Diffuse Glioma (WHO III-IV), NEC
24	M	56	WT	GBM, WHO IV
25	F	36	IDH1-mutiert	Anaplastic Astrocytoma/GBM(WHO III-IV)
26	M	56	WT	Anaplastic Astrocytoma (WHO III)
27	F	69	WT	GBM, WHO IV
28	M	49	WT	GBM, WHO IV
29	F	81	WT	Anaplastic Astrocytoma (WHO III)
30	M	77	WT	GBM, WHO IV
31	F	49	WT	GBM, WHO IV
32	M	70	WT	GBM, WHO IV
33	F	78	WT	GBM, WHO IV
34	M	74	WT	GBM, WHO IV
35	F	76	WT	GBM, WHO IV
36	M	41	WT	GBM, WHO IV
37	F	74	?	GBM, WHO IV
38	M	59	WT	GBM, WHO IV
39	F	69	WT	GBM, WHO IV
